# Biventricular Noncompaction Cardiomyopathy in a Patient Presenting with New Onset Seizure: Case Report

**DOI:** 10.1155/2012/924865

**Published:** 2012-07-11

**Authors:** Oghenerukevwe Odiete, Ramanna Nagendra, Mark A. Lawson, Henry Okafor

**Affiliations:** ^1^Division of Cardiovascular Medicine, Department of Medicine, Vanderbilt University Medical Center, 2220 Pierce Avenue, Preston Research Building, Nashville, TN 37232, USA; ^2^Division of Cardiovascular Medicine, Department of Medicine, Nashville General Hospital, Meharry Medical College, 1005 Dr. D. B. Todd Jr. Boulevard, Nashville, TN 37208, USA

## Abstract

Ventricular noncompaction (VNC) of the myocardium is a rare genetic cardiomyopathy caused by a disorder during endocardial morphogenesis and could be accompanied by life-threatening complications. The major clinical manifestations of VNC are heart failure, arrhythmias, and embolic events. The left ventricle is the most commonly reported affected site, but a few cases of right ventricular involvement have also been reported. We report a case of biventricular noncompaction cardiomyopathy in a 31-year-old woman presenting with a new onset seizure. On the second day of her telemetry-monitored hospitalization, she suffered a witnessed ventricular fibrillation arrest requiring emergency direct-current cardioversion and induced hypothermia. Transthoracic echocardiography (TTE) showed isolated left ventricular (LV) noncompaction and depressed LV systolic function. Subsequent cardiac magnetic resonance imaging (MRI) revealed both left and right ventricular noncompaction. This unusual presentation highlights the importance of a complete and thorough evaluation of patients even when presenting with apparently noncardiac symptom(s).

## 1. Introduction

Ventricular noncompaction (VNC) of the myocardium is a rare genetic cardiomyopathy that is believed to arise from arrested endomyocardial development during embryogenesis [1]. It is a rare idiopathic cardiomyopathy with an incidence of about 0.05% [2, 3]. VNC is classified by the American Heart Association as a primary genetic cardiomyopathy [1] and is characterized by an altered myocardial wall with prominent trabeculae and deep intertrabecular recesses. This results a thickened bilayer of compacted and noncompacted myocardium [4], caused by the arrest of the normal process of endomyocardial morphogenesis. The normal process of trabeculation has been shown to be dependent upon secretion of neuregulin growth factors from the endocardium. Angiogenic factors, such as vascular endothelial growth factor and angiopoietin-1, may also be critical for normal trabeculae formation [5, 6]. The major clinical manifestations of VNC are heart failure, arrhythmias, sudden cardiac death, cardioembolic events, and syncope [7–9]. To our knowledge, seizures have not been previously reported as a manifestation of this condition. Here, we report a case of biventricular noncompaction cardiomyopathy in a young female presenting with new-onset tonic-clonic seizure.

## 2. Case Presentation

A 31-year-old woman presented with a witnessed loss of consciousness while driving. The episode lasted for about five-to-ten minutes and was associated with tonic-clonic seizure-like movement. There was no urinary or bowel incontinence. She had no history of seizures, arrhythmias, or family history of sudden cardiac death.

Physical exam was remarkable only for a 2/6 systolic ejection murmur loudest at the left midsternal border on cardiac auscultation. The laboratory results were within normal limits. Electrocardiogram (ECG) showed sinus rhythm with occasional premature ventricular complexes. An electroencephalogram (EEG) and a head-computed tomography (CT) scan were unrevealing.

**Figure 1:**
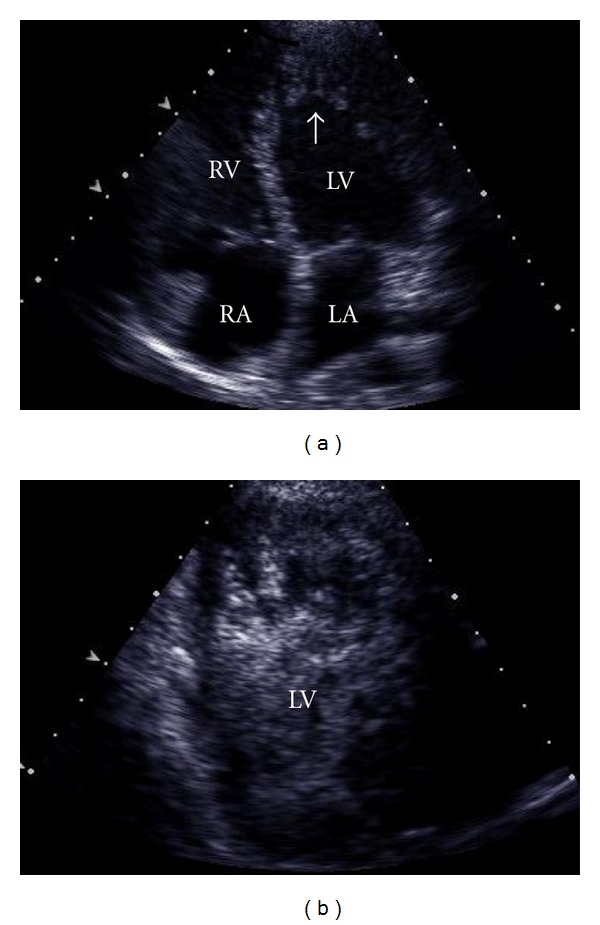
Echocardiogram of the heart. (a) Apical view demonstrating marked trabeculation of the left ventricular apex (white arrow). (b) It shows contrast agent visualized between the ventricular trabeculations. RA: right atrium; LA: left atrium; LV: left ventricle; RV: right ventricle.

**Figure 2:**
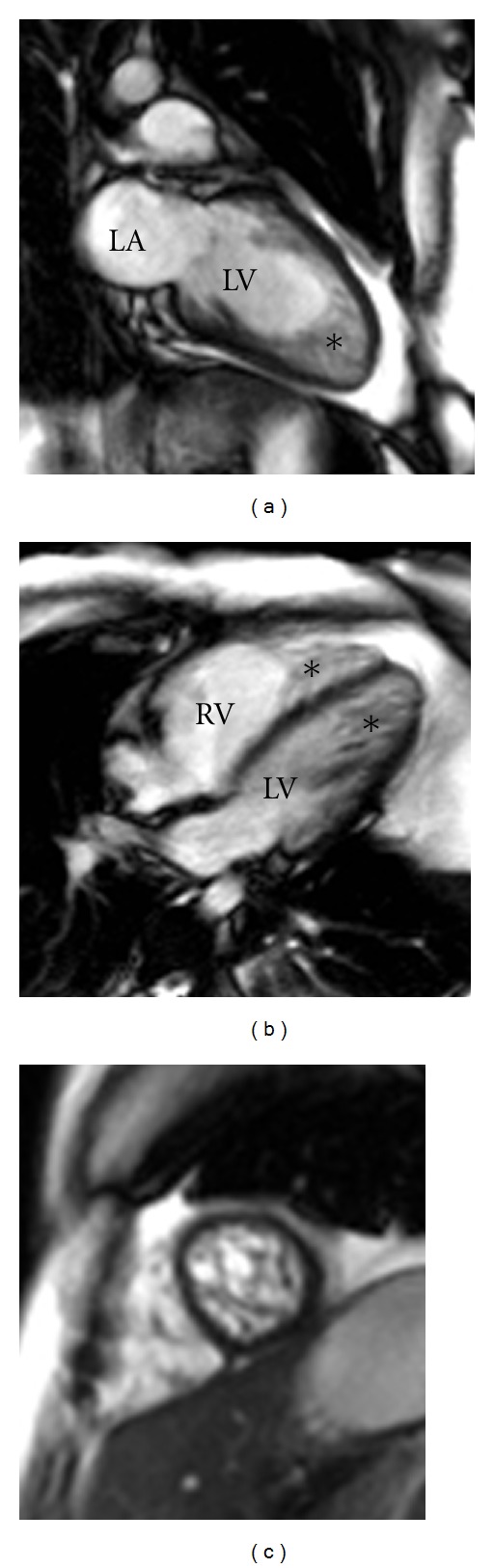
Cardiac MRI of the heart. ((a) and (b)) Cardiac MRI of the LV showing prominent muscular trabeculations (asterisk) and spongiform appearance of the ventricular cavities. (c) Short axis cardiac MRI image through the distal RV and LV, demonstrating trabeculations filling the ventricular cavity.

In the course of the workup, the patient suffered a ventricular fibrillation cardiac arrest requiring defibrillation and induced hypothermia for resuscitation. She recovered without neurologic deficits. Cardiac evaluation with echocardiogram (Figure 1) showed depressed left ventricular systolic function (ejection fraction (EF) of 30–35%), biatrial enlargement, and increased left ventricular (LV) wall thickness. The left ventricular wall showed marked trabeculation within the inner layer of myocardium consistent with LV noncompaction cardiomyopathy. Contrast study showed blood flow through the trabeculated noncompact inner layer to the outer compact layer. Cardiac MRI (Figure 2) demonstrated the prominent LV trabeculation pattern that was noted on TTE, which forms the classic spongiform appearance on the distal one-third of the LV cavity. In addition, an equally prominent trabeculation pattern was seen in the right ventricle (RV) involving the distal one-third of the RV. Since other MRI features of arrhythmogenic RV dysplasia were absent, the MRI findings were consistent with biventricular noncompaction. A single-lead ICD was implanted and the patient was later discharged in stable condition.

## 3. Discussion

VNC of the myocardium is a rare genetic cardiomyopathy. The left ventricle is more frequently involved, but biventricular involvement, as in this case, is rarely encountered [10, 11]. What made our patient unique were not only the individual diagnostic findings, but also the aggregate clinical picture. Oechslin et al.[7] demonstrated in a follow-up study of 34 patients that the most important clinical manifestations of VNC were heart failure (53%), ventricular tachycardia (41%), sudden cardiac death (35%), syncope (18%), and embolic events (24%). Our patient presented solely with a tonic-clonic seizure-like activity and a normal EKG and EEG, but later experienced an in-hospital ventricular fibrillation cardiac arrest. Among all the presenting symptom of VNC, the most common reason for referral was heart failure, [6, 7] with one-third of these patients having NYHA class III/IV at time of diagnosis [12]. Our patient did not have signs or symptoms of heart failure on presentation. However, this does not exclude the fact that she had an asymptomatic cardiomyopathy.

Although tonic-clonic seizure-like activity was the only presenting symptom given the extensive structural heart disease, it is plausible that this patient likely had a ventricular arrhythmic event (since the EEG and ECG were unrevealing) leading to generalized cerebral hypoxia, and thus the tonic-clonic seizure activity. The resulting motor activity has been attributed to a decreased cerebral blood flow [13], which results in generalized cerebral hypoxia and thus mimicking epileptic seizures [13–15]. Furthermore, the abnormal motor activity of syncope due to malignant ventricular arrhythmias [16] can also mimic epileptic seizures. Schott et al. [17] identified cardiac arrhythmias in 20% of the patients referred with idiopathic epilepsy. Patients with VNC are at an increased risk for thromboembolic events [18]. However, in the absence of atria fibrillation or LV systolic dysfunction, the risk for cardioembolic event is rare [19]. Seizure may be an associated complication following an acute stroke [20]. Even though our patient had a negative CT scan of the head, it is conceivable that a thromboembolic event was a plausible cause for the seizure.

Although left ventricular noncompaction is more common, the improved imaging capabilities of cardiac MRI provide opportunity for better characterization of right ventricular involvement. Echocardiography has a crucial role in the diagnosis of VNC, but cardiac MRI, contrast ventriculography, and computed tomography could also be utilized during assessment, especially in patients with poor image quality on echocardiography [21, 22], in order to rule out other cardiac pathological involvement. Borreguero et al. [23] suggested the potential role of cardiac MRI in the evaluation of the RV with noncompacted myocardium. Our patient represents an important example of biventricular noncompaction cardiomyopathy, in which cardiac MRI aided the diagnosis.

In conclusion, VNC is an uncommon disorder accompanied by life-threatening complications. Its unusual presentation highlights the importance of a complete and thorough workup of patients presenting with apparently noncardiac symptom(s). Data is limited on specific therapy for VNC, but it is recommended that medical management be tailored towards the clinical manifestations, and standard guidelines should be applied for patients with reduced LVEF, and heart failure with preserved systolic function [24], in the setting of VNC. Patients with VNC, who meet standard criteria for anticoagulation, should also be managed according to standard guidelines [25]. In addition, periodic holter monitoring may be used to assess the risk for asymptomatic arrhythmias. Finally, patients with VNC should receive implantable cardioverter-defibrillator (ICD) therapy according to standard indications for primary and secondary prevention of sudden cardiac arrest [26, 27].

## References

[1] Maron BJ, Towbin JA, Thiene G (2006). Contemporary definitions and classification of the cardiomyopathies: an American heart association scientific statement from the council on clinical cardiology, heart failure and transplantation committee; quality of care and outcomes research and functional genomics and translational biology interdisciplinary working groups; and council on epidemiology and prevention. *Circulation*.

[2] Richardson P, McKenna RW, Bristow M (1996). Report of the 1995 world health organization/international society and federation of cardiology task force on the definition and classification of cardiomyopathies. *Circulation*.

[3] Hughes ML, Carstensen B, Wilkinson JL, Weintraub RG (2007). Angiographic diagnosis, prevalence and outcomes for left ventricular noncompaction in children with congenital cardiac disease. *Cardiology in the Young*.

[4] Weiford BC, Subbarao VD, Mulhern KM (2004). Noncompaction of the ventricular myocardium. *Circulation*.

[5] Zambrano E, Marshalko SJ, Jaffe CC, Hui P (2002). Isolated noncompaction of the ventricular myocardium: clinical and molecular aspects of a rare cardiomyopathy. *Laboratory Investigation*.

[6] Ritter M, Oechslin E, Sütsch G, Attenhofer C, Schneider J, Jenni R (1997). Isolated noncompaction of the myocardium in adults. *Mayo Clinic Proceedings*.

[7] Oechslin EN, Attenhofer Jost CH, Rojas JR, Kaufmann PA, Jenni R (2000). Long-term follow-up of 34 adults with isolated left ventricular noncompaction: a distinct cardiomyopathy with poor prognosis. *Journal of the American College of Cardiology*.

[8] Duru F, Candinas R (2000). Noncompaction of ventricular myocardium and arrhythmias. *Journal of Cardiovascular Electrophysiology*.

[9] Murphy RT, Thaman R, Blanes JG (2005). Natural history and familial characteristics of isolated left ventricular non-compaction. *European Heart Journal*.

[10] Ulusoy RE, Kucukarslan N, Kirilmaz A, Demiralp E (2006). Noncompaction of ventricular myocardium involving both ventricles. *European Journal of Echocardiography*.

[11] Cavusoglu Y, Ata N, Timuralp B (2003). Noncompaction of the ventricular myocardium: report of two cases with bicuspid aortic valve demonstrating poor prognosis and with prominent right ventricular involvement. *Echocardiography*.

[12] Jenni R, Oechslin EN, Van Der Loo B (2007). Isolated ventricular non-compaction of the myocardium in adults. *Heart*.

[13] Zaidi A, Clough P, Cooper P, Scheepers B, Fitzpatrick AP (2000). Misdiagnosis of epilepsy: many seizure-like attacks have a cardiovascular cause. *Journal of the American College of Cardiology*.

[14] Sander JWAS, O’Donoghue MF (1997). Epilepsy: getting the diagnosis right. *British Medical Journal*.

[15] Linzer M, Grubb BP, Ho S, Ramakrishnan L, Bromfield E, Estes NAM (1994). Cardiovascular causes of loss of consciousness in patients with presumed epilepsy: a cause of the increased sudden death rate in people with epilepsy?. *American Journal of Medicine*.

[16] Aminoff MJ, Scheinman MM, Griffin JC, Herre JM (1988). Electrocerebral accompaniments of syncope associated with malignant ventricular arrhythmias. *Annals of Internal Medicine*.

[17] Schott GD, McLeod AA, Jewitt DE (1977). Cardiac arrhythmias that masquerade as epilepsy. *British Medical Journal*.

[18] Pitta S, Thatai D, Afonso L (2007). Thromboembolic complications of left ventricular noncompaction: case report and brief review of the literature. *Journal of Clinical Ultrasound*.

[19] Stöllberger C, Blazek G, Dobias C, Hanafin A, Wegner C, Finsterer J (2011). Frequency of Stroke and Embolism in Left Ventricular Hypertrabeculation/Noncompaction. *American Journal of Cardiology*.

[20] Goswami RP, Karmakar PS, Ghosh A Early seizures in first-ever acute stroke patients in india: incidence, predictive factors and impact on early outcome.

[21] Harada T, Ohtaki E, Kitahara K, Sumiyoshi T, Hosoda S (2002). Carvedilol-induced changes in cardiac diastolic performance in a patient with isolated noncompaction of the myocardium. *Internal Medicine*.

[22] Hamamichi Y, Ichida F, Hashimoto I (2001). Isolated noncompaction of the ventricular myocardium: ultrafast computed tomography and magnetic resonance imaging. *International Journal of Cardiovascular Imaging*.

[23] Borreguero LJ, Corti R, de Soria RF, Osende JI, Fuster V, Badimon JJ (2002). Images in cardiovascular medicine. Diagnosis of isolated noncompaction of the myocardium by magnetic resonance imaging.. *Circulation*.

[24] Hunt SA, Abraham WT, Chin MH (2009). 2009 focused update incorporated into the ACC/AHA 2005 guidelines for the diagnosis and management of heart failure in adults: a report of the american college of cardiology foundation/american heart association task force on practice guidelines: developed in collaboration with the international society for heart and lung transplantation. *Circulation*.

[25] Stöllberger C, Blazek G, Dobias C, Hanafin A, Wegner C, Finsterer J (2011). Frequency of stroke and embolism in left ventricular hypertrabeculation/noncompaction. *American Journal of Cardiology*.

[26] Zipes DP, Camm AJ, Borggrefe M (2006). Acc/aha/esc 2006 guidelines for management of patients with ventricular arrhythmias and the prevention of sudden cardiac death: a report of the american college of cardiology/american heart association task force and the european society of cardiology committee for practice guidelines (writing committee to develop guidelines for management of patients with ventricular arrhythmias and the prevention of sudden cardiac death). *Journal of the American College of Cardiology*.

[27] Epstein AE, Dimarco JP, Ellenbogen KA (2008). Acc/aha/hrs 2008 guidelines for device-based therapy of cardiac rhythm abnormalities: a report of the american college of cardiology/american heart association task force on practice guidelines (writing committee to revise the acc/aha/naspe 2002 guideline update for implantation of cardiac pacemakers and antiarrhythmia devices): developed in collaboration with the american association for thoracic surgery and society of thoracic surgeons. *Circulation*.

